# Prospective observational study evaluating acute and delayed treatment related toxicities of prophylactic extended field volumetric modulated arc therapy with concurrent cisplatin in cervical cancer patients with pelvic lymph node metastasis

**DOI:** 10.1016/j.tipsro.2021.02.009

**Published:** 2021-03-09

**Authors:** N. Ballari, B. Rai, A. Bahl, B.R. Mittal, S. Ghoshal

**Affiliations:** a Departments of Radiotherapy, Postgraduate Institute of Medical Education and Research (PGIMER), Chandigarh, India; b Nuclear Medicine, Postgraduate Institute of Medical Education and Research (PGIMER), Chandigarh, India

**Keywords:** Pelvic lymph node positive locally advanced cervical cancer, Prophylactic para aortic extended field SIB-VMAT, Grade 2 and above Acute G.I or GU Toxicity, Chronic toxicity, Improved therapeutic Index

## Abstract

**Purpose:**

To evaluate the treatment related acute and delayed toxicities of extended field Volumetric modulated arc therapy (VMAT) with concurrent chemotherapy in patients of locally advanced cervical cancer with pelvic lymph nodes.

**Material and methods:**

From 2014 to 2016, 15 patients of locally advanced cervical cancer with Fluoro-deoxyglucose positron emission tomography (FDG-PET) positive pelvic lymph nodes were treated with extended field Simultaneous integrated boost (SIB)-VMAT 45 Gy/55 Gy/25#/5weeks and concurrent cisplatin. Acute toxicities were documented according to common terminology criteria for adverse events version 4 (CTCAE v.4). Dose volume parameters and patient characteristics were analyzed for association with toxicities.

**Results:**

Median age of patients at diagnosis was 48 years. 40% (6 patients) were stage IIB & 60% (9 patients) were stage IIIB. Median number of involved pelvic lymph nodes was 2 (range, 1–4), commonest location was external iliac lymph node region (86%). Median number of concurrent chemotherapy cycles received was five. Treatment was well tolerated and there were no grade ≥ 3 acute toxicities. Commonest acute toxicities observed were vomiting (≥grade2 −13.3%) followed by & nausea (grade ≥ 2 in 6%) and were associated with volume of bowel bag receiving 45 Gy. Constitutional symptoms (≥grade 2) were observed in 6% patients and had no dosimetric associations. At a median follow up of 43 months, delayed ≥ grade1, 2, 3 toxicity were observed in 80%, 0%, and 0% respectively with diarrhea being the commonest.

**Conclusion:**

Prophylactic para aortic extended field VMAT with concurrent chemotherapy for locally advanced cervical cancer is well tolerated with acceptable acute toxicity profile. Significant grade 3 acute/delayed toxicities were not observed in this cohort of patients.

## Highlights

• Para aortic region harbors occult disease in lymph node positive cervical cancer.
• Extended field RT may improve outcomes in these patients at the risk of toxicity.
• VMAT/IMRT has been shown to reduce acute toxicities in pelvic malignancies.
• There exists a paucity of data on acute treatment related toxicity with EF-VMAT.
• Extended field VMAT in our study was well tolerated with no grade 3 toxicity.
• No grade ≥2 delayed toxicity was reported at a median follow up of 43 months.

## Introduction

Cervical cancer is the second commonest malignancy diagnosed among Indian women, [1], [2]. Radical pelvic chemo-radiation followed by brachytherapy has produced 5 year overall survival rates, nodal and systemic control rates of 89, 87% and 77% respectively (all stages). After chemo radiation, most failures occur in para aortic region (69%) and involvement of pelvic lymph nodes at diagnosis was the strongest predictor of para aortic lymph node recurrences [3], [4], [5]. Thus prophylactic irradiation of the para aortic region in cervical cancer was hypothesized to reduce para aortic failures and overall treatment results [6], [7]. However, with the use of conventional 2 dimensional radiation techniques, therapeutic index could not be maintained due to treatment related toxicities - acute and chronic bowel toxicities of 8% with extended field RT (radiotherapy)compared to 4% in pelvic EBRT(External beam radiotherapy) [8], [9], [10], [11], [12].

**Fig. 1:**
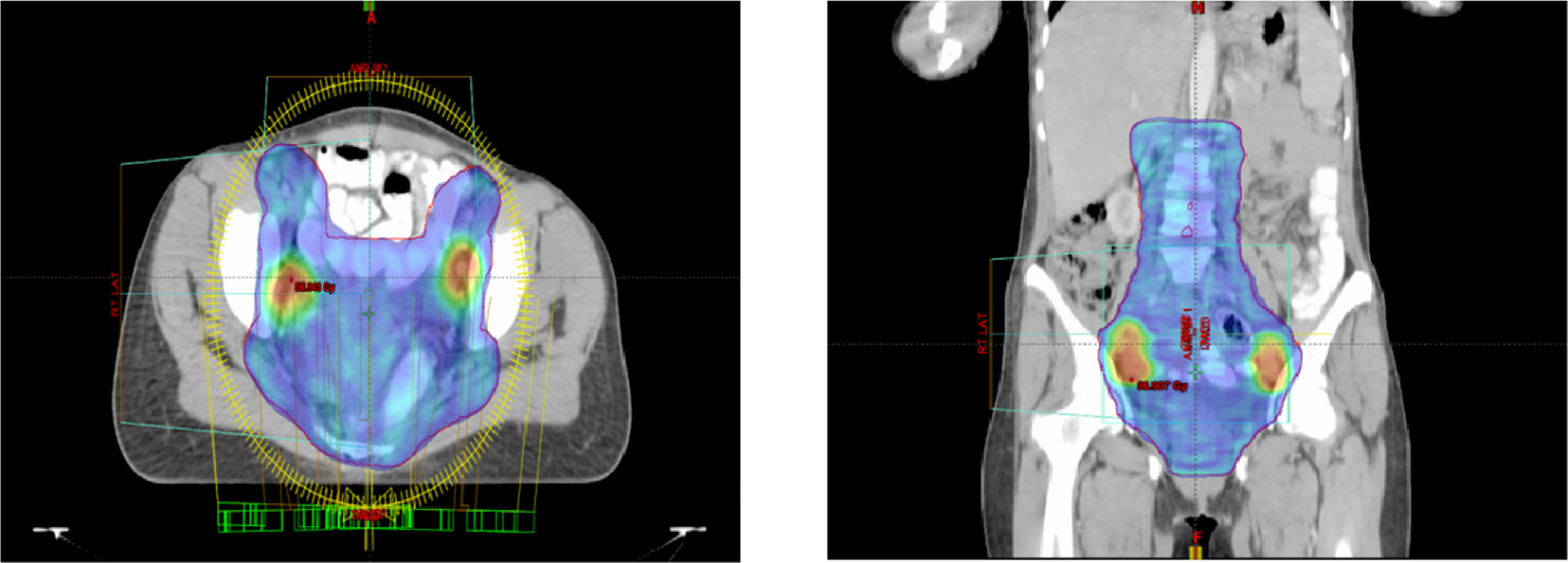
Pelvic RT volume and SIB volume.

The evolution of radiation techniques continued over the past two decades and now with the availability of highly conformal radiation delivery techniques like Volumetric Modulated Arc Therapy (VMAT), it is now possible to achieve a favorable therapeutic index by limiting the doses to organs at risk [13], [14], [15], [16], [17], [18], [19]. However, detailed profiles of acute and delayed treatment related toxicity with this approach are scarcely reported in literature. The purpose of this study was to prospectively record the acute and delayed treatment related toxicities in patients with pelvic node positive cervical cancer undergoing prophylactic extended field volumetric modulated arc radiotherapy and concurrent chemotherapy (see Fig. 1).

## Material and methods

The study was designed as a single arm prospective observational study and was conducted between August 2014- March 2016 in the department of radiotherapy and oncology, PGIMER, Chandigarh, India. A convenient sample size of 15 patients was chosen. Eligibility criteria for enrolment into the study were biopsy confirmed FIGO (International federation of gynecology and obstetrics) (2009) [36] stage IIB-IIIB cervical cancer, positive pelvic lymph nodes and negative para aortic nodes on FDG PET-CT (Fluoro-deoxyglucose positron emission tomography computed tomography), Karnofsky performance scale >70 [34], normal creatinine clearance (>80 ml/min). Uncontrolled medical co-morbidities, previous history of chemo-radiation to the pelvis and postoperative status were exclusion criteria. Informed consent was taken from all patients. Institutional ethics committee approval was obtained for this study. Pre-treatment workup included a pelvic examination followed by complete staging workup with complete haemogram, liver function tests, kidney function tests, chest X-ray, ECG (Electrocardiogram) and whole body FDG PET-CT. The FDG uptake in the para-aortic and pelvic lymph nodes, if greater than the mediastinal blood pool activity, was taken as positive for pelvic or para aortic metastases. Cystoscopy and proctoscopy were advised only in suspicious cases to rule out bladder and rectum infiltration respectively. All patients received EBRT dose of 45 Gy in 25 fractions over 5 weeks, at 1.8 Gy per fraction to pelvic and para-aortic regions, with a simultaneous integrated boost of 55 Gy in 25 fractions over 5 weeks at 2.25 Gy per fraction to the involved pelvic nodes. Concurrent chemotherapy was given to all patients with weekly injection Cisplatin at 40 mg/m^2^. Intra-cavitary brachytherapy was given after completion of external radiotherapy to a dose of 9 Gy HDR (High-dose-rate) prescribed to point A in two fractions one weekapart.

### External beam radiotherapy planning

Patient was given laxative from two days prior to the scan day to have an empty rectum. The patients were advised to take one liter of water mixed with 20 ml of gastrograffin oral contrast within 2 h before the image acquisition and were asked to void completely. After this, patients were made to consume 500 ml of plain water. Rectal contrast was given by dissolving 20 ml of gastrograffin in 50 ml of normal saline. CT (computed tomography) scan was acquired 15 min later. Injection iohexol 100 m l was used as IV contrast abiding by the cross’s method of intravenous contrast administration. The same rectum and bladder protocol were maintained during daily treatment.

Planning computed tomography scan of pelvis and abdomen was acquired in supine position using a footrest as a positioning device was acquired on a Philips (Amsterdam, Netherlands) CT scanner (Brilliance big bore) at 2.5 mm slice thickness from the level of diaphragm till the upper third of femur.

Pelvic treatment volume delineation was in accordance with published guidelines [23], [24], [25]. For delineation of the prophylactic para aortic nodal basin, great vessels were delineated as surrogates, and the space between the psoas muscles was included in accordance with guidelines given by Beriwal et al. [26]. Bladder, rectum, sigmoid, femurs, kidneys and spinal cord were delineated as organs at risk (OAR)according to RTOG (Radiotherapy oncology group) guidelines, and all the potential space for bowel loops in the abdomen was delineated as bowel bag as per Portelance et al. [13].

Planning dose volume constraints for the PTV (Planning target volume) and OAR volumes, as presented in Table 1, were followed and Non bone marrow sparing VMAT plans were generated on eclipse planning system version 11.

**Table 1:** . Dose volume constraints.

Structure	Planning constraint
PTV final	95% of PTV to receive 95% of 45 Gy/25#/5 weeks 1% PTV to receive 115% of 45 Gy/25#/5 weeks
PTV SIB	100% of PTV SIB(Simultaneous integrated boost) to receive 100% of 55 Gy/25#/5 weeks
CTV(clinical target volume)	At least 95% of CTV to receive 100% of prescription dose
Bowel	V45 < 195 cc, D-max < 50 Gy (maximum dose)
Rectum	V40 < 40% Dmax < 50 Gy
Bladder	V40 < 40% Dmax < 50 Gy
Bilateral kidneys	25% of bilateral kidneys - < 16 Gy Mean dose to B/L kidneys- <18 Gy
Femoral heads	Dmax < 46 Gy
Spinal cord	D-max < 45 Gy

### Concurrent chemo-radiation

All the patients were treated on Varian Trilogy linear accelerator (Varian medical systems, Palo Alto CA). On board treatment verification was done using daily Kilo voltage imaging and biweekly cone beam CT imaging. Pelvic bones and body of vertebra were matched while using KV orthogonal image verification daily. On the days where CBCT (cone beam computed tomography) was used, both bone to bone and SIB volume match was performed to ensure accurate treatment delivery. All patients received weekly inj. cisplatin starting from the day of treatment) at a dose of 40 mg/m^2^ once a week for 5 weeks. Chemotherapy was delayed if the total leukocyte count was <3000/mm^3^ or the peripheral platelet count was <100000/mm^3^.

### Acute toxicity evaluation

All patients while on external beam radiotherapy underwent weekly evaluation with complete blood counts, kidney function tests. Baseline and on treatment acute toxicities namely constitutional, hematological, bowel, genitourinary, were graded in accordance with CTCAE v4.03. A toxicity chart was custom made for the above and shown in appendix 2 (Common Terminology Criteria for Adverse Events) [35].

### Response evaluation and follow up

First follow up of patients was at 6 weeks of completion of external beam radiotherapy and brachytherapy. A clinical examination and toxicity assessment using same toxicity chart as the one used during the course of radiotherapy was done. If the patients were found clinically disease free, they were followed up at three monthly intervals as per departmental protocol. A PET- CT was repeated six months after treatment completion to assess treatment response. RECIST (response evaluation criteria in solid tumors) criteria were followed for response assessment. A clinical examination and delayed toxicity evaluation which included CTCAE v4 grading was recorded at each visit.

### Statistics

Patient details like age, stage, histology, tumor size, lymph node size and number, baseline investigations, acute and delayed toxicities, dosimetric parameters were entered in Statistical package for SPSS v 17 (Statistical Package for the Social Sciences) for statistical analysis. Descriptive as well as frequency distributions of all parameters were obtained. The primary end point of the study was to assess the acute toxicity profile of extended field SIB-VMAT. The secondary endpoints of the study were to look for associations between acute toxicities with dosimetric parameters using Uni-variate and multivariate analysis and to record delayed toxicities. A ‘p’ value < 0.05 was considered statistically significant.

## Results

### Demographic data

Descriptive statistics were used to describe demographic details and are presented in Table 2. Uni-variate and multivariate analysis were done to look for associations of > grade 2 toxicities with various treatment related parameters. The median time taken for completion of external beam radiation therapy was 5.2 weeks. Median time for treatment completion was 9.8 weeks (6.4–13.7 weeks). Median number of concurrent chemotherapy cycles was 5. There were no interruptions in radiotherapy due to acute toxicities.

**Table 2:** . Demographic data.

Characteristics	Proportion
**Median age (range)**	48 (range 39–60)
**Squamous cell histology**	13/15 (86%)
**FIGO stage** IIB III B	6 9
**Median primary tumour size cm(range**)	5.8 cm (range 1.8–9.3 cm)
**Median primary tumor SUV max (range)**	20.6 (range 8.10–28.0)
**Site of lymph node involvement** Unilateral external iliac Bilateral external iliac Multiple sites within the pelvis	26.7% (7) 60% (18) 13.3% (4)
**Median no of lymph nodes involved (range)**	2(range 1.0–4.0)
**Median size of the largest lymph nodes(range)**	1.6 cm (range 1.1–3.3)
**Median lymph node SUV maximum(range)**	5.6 (range 3.6–18.5)
**No: of lymph nodes subjected to SIB**	22

### Dosimetric analysis

The median volume of PTV in the patient cohort was 2451 cc (range 2061–2636 cc). The dosimetric parameters of PTV and OARS are summarized in the Table 3.

**Table 3:** . Dosimetric profile of the study group.

Dosimetric parameter	Median values
PTV volume	2451 cc
PTV D2	115%
PTV D50	100.13%
PTV D98	95.3%
PTV SIB volume	33 cc
PTV SIB D2	103.47%
PTV SIB D50	95.14%
PTV SIB D98	97.25%
BOWEL BAG VOLUME	3631 cc
V45 BOWEL	142.54 cc
RECTUM Dmax	48.01 Gy
BLADDER Dmax	48.01 Gy
KIDNEYS DMEAN	15.63 Gy
FEMORAL HEAD Dmax	45.21 Gy
SPINAL CORD Dmax	42.87 Gy

### Toxicity

**Fig. 2:**
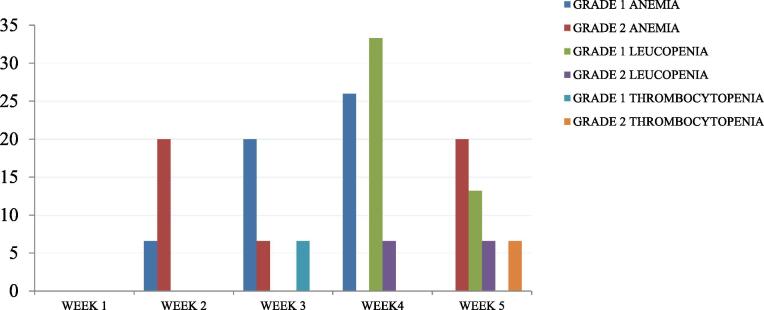
Hematological toxicity over time during EBRT.

Weekly toxicity evaluation was done and the highest reported toxicity grade from the date of EBRT initiation to 6 weeks after treatment completion was recorded as worst acute toxicity and is presented in Table 4. EF-VMAT was well tolerated with predominant occurrence of Grade 1 and 2 toxicities. There was no grade 3 anemia or leucopenia in the study group, no blood transfusions were warranted and there were no RT interruptions because of acute toxicity. Two patients in the group needed chemotherapy deferral owing to grade 2 leucopenia (13.3%). The profile of hematological toxicity over treatment time is presented in Fig. 2. The median time for development of ≥ grade 2 anemia was week 5 (range 2–5), for ≥ grade 2 leucopenia this was week 4. Dosimetric parameters and acute toxicities were subjected to multivariate analyses. Thrombocytopenia was associated with increase in overall treatment time. The onset of pain abdomen was earlier with increasing number of chemotherapy cycles. The occurrence of vomiting as a toxicity increased with increase in the volume of bowel bag receiving 45 Gy (p < 0.0021). No increased acute toxicities were noted because of presence of SIB volumes.

**Table 4:** . Acute toxicity.

Acute toxicity	Grade	Proportion of patients (n)
*Constitutional symptoms*		
Fatigue	0 1 2 3	13.3%(2/15) 80%(12/15) 6%(1/15) 0
Fever	0 1 2 3	73.3%(11/15) 26.6%(4/15) 0 0
Weight loss	0 1 2 3	73.3%(11/12) 33.3%(5/12) 0 0
*Haematological*		
Anemia	1 2 3	53%(8/15) 47% (7/15) 0
Neutropenia	0 1 2	40%(6/15) 47%(7/15) 14%(2/15)
Thrombocytopenia	0 1 2	87%(13/15) 6%(1/15) 6%(1/15)
*Gastrointestinal*		
Pain abdomen	0 1 2 3	13.3%(2/15) 67%(10/15) 13.3%(2/15) 6% (1/15)
Anorexia	0 1 2 3	40%(6/15) 53.3%(8/15) 6%(1/15) 6%(1/15)
Dyspepsia	0 1 2 3	33.3%(5/15) 60%(9/15) 6%(1/15) 0
Nausea	0 1 2 3	20%(3/15) 73.3%(11/15) 6%(1/15) 0
Vomiting	0 1 2 3	40%(6/15) 46.6%(7/15) 13.3%(2/15) 0
Diarrhea	0 1 2 3	46.6%(7/15) 6%(1/15) 13.3%(2/15) 0
Constipation	0 1 2 3	66.6%(10/15) 33.3%(5/15) 0 0
Proctitis	0 1 2 3	73.3%(11/15) 26.6%(4/15) 0 0
**Genitourinary** Cystitis, Frequency, Incontinence, Retention.	0 1 2 3	94% (14/15) 6% (1/15) 0 0
**Radiation dermatitis**	0 1 2 3	6%(1/15) 80%(12/15) 13.3%(2/15) 0

### Delayed toxicity and clinical response

The median follow up period for the cohort was 43 months (range 6–58 months). Delayed toxicity predominantly manifested as grade1 pain abdomen which was seen in 86% (13/15) of the patients and there was isolated delayed grade1 cystitis in the patient cohort 6.7% (1/15). There was no > grade 2 delayed toxicity of any category. The median follow up was 43 months (range 6–57 months).

At first follow up, 11/15 patients had complete clinical response. Follow up PET CT at 6 months revealed persistent local disease in 3 patients and pelvic nodal disease in 1 patient. At median follow up, 12 patients were alive, while 8 had CR, 7 had local disease, 4 patients had pelvic nodal disease, and 4 developed distant metastasis. Isolated PA nodal recurrences were not observed in any patient. The median time to recurrence was 12 months (2–31 months). No patient had local failure as their only site of recurrence.

## Discussion

The benefit of prophylactic extended field radiotherapy along with concurrent chemotherapy has long been hypothesized and the techniques to test this have evolved over the past three decades, starting off with the use of conventional technique for radiation delivery in the late 90′s and early 2000′s to the present day where IMRT is a routine practice. Adoption of a new target delineation scheme for para aortic region lymphatics coupled with conformal radiation therapy techniques has led to a drastic decline in grade 3 acute toxicities [13], [14], [15], [16], [17], [18], [19], [20], [21] confirmed again by the results of our study. A retrospective analysis conducted at our institute included patients of locally advanced cervical cancer patients treated with pelvic 3DCRT and concurrent cisplatin revealed that the majority of patients had grade 2 and below acute toxicities. The incidence of grade 3 vomiting, diarrhea &hematological toxicities was 5.2%, 5.7% and 1.4% respectively [29]. There were treatment interruptions in 8 patients due to grade 3 diarrhoea. The findings of present study stand superior in the fact that there were no grade 3 acute toxicities or treatment interruptions.

Studies in the past using conventional 4 field technique to deliver extended field radiation therapy resulted in grade 3 and above acute bowel toxicities in the range of 2–25% and grade 3 and above hematologic toxicities in the range of 20–80% [8], [9], [10], [11], [12]. A few studies attempting EF-IMRT and concurrent chemotherapy in LACC showed an incidence of grade 3 hematological toxicity in the range of 19–28% [20], [21]. Despite using a non-bone marrow sparing approach and large volume of irradiation in the present study, we did not see any grade 3 hematological toxicities and treatment breaks because of them.

A favorable acute bowel toxicity profile was seen in our cohort of patients. The planning constraint to limit the volume of bowel bag receiving 45 Gy to less than 195 cc was the main reason for this. It was seen that compared to a group of stage and age matched patients who received pelvic 3DCRT at our institute in the past, with EF-VMAT planning we could reduce the volume of bowel bag receiving 45 Gy by 71%. This reduction in volume of irradiated bowel to a dose of 45 Gy was more when compared to the observations published by Mundt et al where they showed that the volume of bowel bag receiving 45 Gy was reduced by 50% with the use of pelvic IMRT compared to pelvic 3DCRT [22].

Acute grade 3 vomiting occurred very commonly with conventional extended field chemo-radiotherapy and ranged from 18.6% to 25 % across various studies [9], [10]. We did not encounter any grade 3 vomiting in our study group [9]. Even with extended field IMRT and concurrent chemotherapy reported grade 2 and above nausea and vomiting was the range of 21–61 % [15], [16], [17], [18]. Compared to these studies, the incidence of grade 2 and above nausea and vomiting was lower in our study group (13.3% vs. 25%) [20], [21]. Grade 3 pain abdomen was the most serious bowel toxicity and occurred only in one patient. The incidence of grade 2 diarrhoea was 13.3% compared to studies which used conventional extended field radiotherapy with concurrent chemotherapy which have reported grade 2 or higher diarrhoea to the tune of 40–50% [8], [9], [10], [11], [12].

The median dose of chemotherapy received by our patients in the study group was 265 mg and median number of chemotherapy cycles was 5. There was no association between toxicities and chemotherapy dose. This is in agreement with previous concurrent chemo-radiation trials, where standard dose of 40 mg/m^2^ were used and no correlation between chemotherapy dose and toxicities was observed [3].

Despite the use of a SIB to boost the involved pelvic lymph nodes, there wasn’t any increase in acute toxicities attributable to the volume of high dose regions surrounding the SIB. This finding is consistent with those in the study by Gerstzen et al and Vargo et al where they used a similar dose to boost involved pelvic lymph nodes. They reported that SIB was well tolerated and did not report any increased toxicity due to SIB [15], [19]. Multivariate analysis showed a negative association between the volume of bowel bag receiving 45 Gy and the incidence of vomiting and pain abdomen. Thus our results suggest that VMAT is successful in producing a dosimetric advantage that translates into a clinical reduction in acute toxicity.

The benefit of limiting treatment related acute toxicities spills over to result in reduction of chronic toxicities as suggested in the past [32]. All patients avoided chronic bowel, bladder and constitutional toxicities“.

IMRT has significantly reduced the incidence of CTCAE V3 grades 1, 2, 3 diarrhoea/ EORTC very much diarrhoeaRecently it has been identified that V43 Gy and para aortic irradiation determined the incidence of late diarrhea [30]. Similarly, none of our patients in the study group had chronic diarrhoea.

On board image verification is an essential ultimate step in accurate treatment delivery of highly conformal radiotherapy techniques like SIB VMAT. This can affect both treatment results as well as toxicity outcome. Our image guidance protocol based on daily kV imaging and supported by biweekly CBCT imaging enabled us to formulate margin recommendations when simultaneously boosting pelvic nodes in cervical cancer [31].

Local site was the most common site of failure in our group (46%). Pelvic lymph node control was 73% and para aortic control was 86%. We found that prophylactic para aortic RT reduced the incidence of para aortic lymph node failures as well as distant metastasis when compared to pelvic RT alone. In the Embrace cohort, Nomden et al analyzed pelvic lymph node status at diagnosis and the patterns of failures [5]. It was seen that almost half of the cohort had pelvic lymph node involvement at diagnosis and elective radiation to para aortic region in these patients reduced failure rate to 7% in patients with documented pelvic lymph nodes [5]. A Cochrane review of studies which tested EF-RT showed improved para aortic region and distant control when compared to pelvic RT alone. However, the most common site of recurrence was loco-regional like in our study [27].

The reasons for local failure in our subset of patients may be multiple. We enrolled patients with involved pelvic lymph nodes which according to the current FIGO staging have been upstaged to a distinct stage IIIc1 considering the fact that these are the patients with poorer outcomes irrespective of tumor size and status of parametrium. Since almost all of our patients had large size tumors and bulky parametrium involvement, probably the inadequate brachytherapy dose led to an increase in local failures. Perhaps with image guided brachytherapy and incorporation of combined interstitial brachytherapy, dose escalation as shown in embrace and retro Embrace studies, these tumors with larger than usual volumes can be treated with a higher dose [28]. Experience from the past studies shows a local control for patients with locally advanced cervical cancer with radical chemo radiation ranging from 60 to 80% and 5 year overall survival of 30–60%.(Stage II & III) and results of our study confer with them. There were also distant failures highlighting the need for adjuvant chemotherapy in this subset of patients. Findings from the OUTBACK trial are expected to answer this question [33].

The main shortcoming of our study was the sample size. A larger sample size and longer median follow up would probably be able to answer these questions. A quality of life assessment conducted at baseline and during subsequent follow up visits would have quantified the improvement in reduction of low grade, yet niggling late toxicities.

## Conclusions

Para aortic irradiation either prophylactic or therapeutic is now a recommended treatment modality for cervical cancer stage IIIc1 and IIIc2 patients and is being practiced in the ongoing EMBRACE II protocol [37]. In this context, data regarding acute toxicity profile is imperative. The results of our study prove that we were successful in avoiding > grade 2 acute toxicities and delayed toxicities. Thus, the theoretical benefit of extended field radiotherapy and concurrent chemotherapy with acceptable acute toxicities locally advanced cervical cancer can now be realized with VMAT.

## Declaration of Competing Interest

The authors declare that they have no known competing financial interests or personal relationships that could have appeared to influence the work reported in this paper.

## Figures and Tables

**Table:** 

Date of RT initiation	Baseline	Week 1	Week 2	Week 3	Week 4	Week 5	End of EBRT	6 weeks after ICBT
*Hematological*								
Hemoglobin								
TLC								
Platelets								

*Constitutional*								
Fatigue								
Weight loss								
Fever								

*Gastrointestinal*								
Anorexia								
Nausea								
Vomiting								
Diarrhea								
Constipation								
Proctitis								

Genitourinary								
Frequency								
Urgency								
Retention								
Cystitis								
SKIN								
Radiation dermatitis								

**Table:** 

	Item No	Recommendation	Page No
**Title and abstract**	1	(*a*) Indicate the study’s design with a commonly used term in the title or the abstract	1
		(*b*) Provide in the abstract an informative and balanced summary of what was done and what was found	1

**Introduction**			
Background/rationale	2	Explain the scientific background and rationale for the investigation being reported	2
Objectives	3	State specific objectives, including any prespecified hypotheses	2

**Methods**			
Study design	4	Present key elements of study design early in the paper	2
Setting	5	Describe the setting, locations, and relevant dates, including periods of recruitment, exposure, follow-up, and data collection	2
Participants	6	(a) *Cohort study*—Give the eligibility criteria, and the sources and methods of selection of participants. Describe methods of follow-up *Case-control study*—Give the eligibility criteria, and the sources and methods of case ascertainment and control selection. Give the rationale for the choice of cases and controls *Cross-sectional study*—Give the eligibility criteria, and the sources and methods of selection of participants	2
		(b) *Cohort study*—For matched studies, give matching criteria and number of exposed and unexposed *Case-control study*—For matched studies, give matching criteria and the number of controls per case	
Variables	7	Clearly define all outcomes, exposures, predictors, potential confounders, and effect modifiers. Give diagnostic criteria, if applicable	3
Data sources/ measurement	8*	For each variable of interest, give sources of data and details of methods of assessment (measurement). Describe comparability of assessment methods if there is more than one group	5
Bias	9	Describe any efforts to address potential sources of bias	
Study size	10	Explain how the study size was arrived at	2
Quantitative variables	11	Explain how quantitative variables were handled in the analyses. If applicable, describe which groupings were chosen and why	
Statistical methods	12	(*a*) Describe all statistical methods, including those used to control for confounding	5
		(*b*) Describe any methods used to examine subgroups and interactions	
		(*c*) Explain how missing data were addressed	
		(*d*) *Cohort study*—If applicable, explain how loss to follow-up was addressed *Case-control study*—If applicable, explain how matching of cases and controls was addressed *Cross-sectional study*—If applicable, describe analytical methods taking account of sampling strategy	
		(*e*) Describe any sensitivity analyses	

**Table:** 

Results			
Participants	13*	(a) Report numbers of individuals at each stage of study—eg numbers potentially eligible, examined for eligibility, confirmed eligible, included in the study, completing follow-up, and analysed	5
		(b) Give reasons for non-participation at each stage	
		(c) Consider use of a flow diagram	
Descriptive data	14*	(a) Give characteristics of study participants (eg demographic, clinical, social) and information on exposures and potential confounders	5
		(b) Indicate number of participants with missing data for each variable of interest	
		(c) *Cohort study*—Summarise follow-up time (eg, average and total amount)	8
Outcome data	15*	*Cohort study*—Report numbers of outcome events or summary measures over time	
		*Case-control study—*Report numbers in each exposure category, or summary measures of exposure	
		*Cross-sectional study—*Report numbers of outcome events or summary measures	
Main results	16	(*a*) Give unadjusted estimates and, if applicable, confounder-adjusted estimates and their precision (eg, 95% confidence interval). Make clear which confounders were adjusted for and why they were included	6,7
		(*b*) Report category boundaries when continuous variables were categorized	
		(*c*) If relevant, consider translating estimates of relative risk into absolute risk for a meaningful time period	
Other analyses	17	Report other analyses done—eg analyses of subgroups and interactions, and sensitivity analyses	5

*Discussion*			
Key results	18	Summarise key results with reference to study objectives	9
Limitations	19	Discuss limitations of the study, taking into account sources of potential bias or imprecision. Discuss both direction and magnitude of any potential bias	10
Interpretation	20	Give a cautious overall interpretation of results considering objectives, limitations, multiplicity of analyses, results from similar studies, and other relevant evidence	10
Generalisability	21	Discuss the generalisability (external validity) of the study results	11

*Other information*			
Funding	22	Give the source of funding and the role of the funders for the present study and, if applicable, for the original study on which the present article is based	

**Figure:**
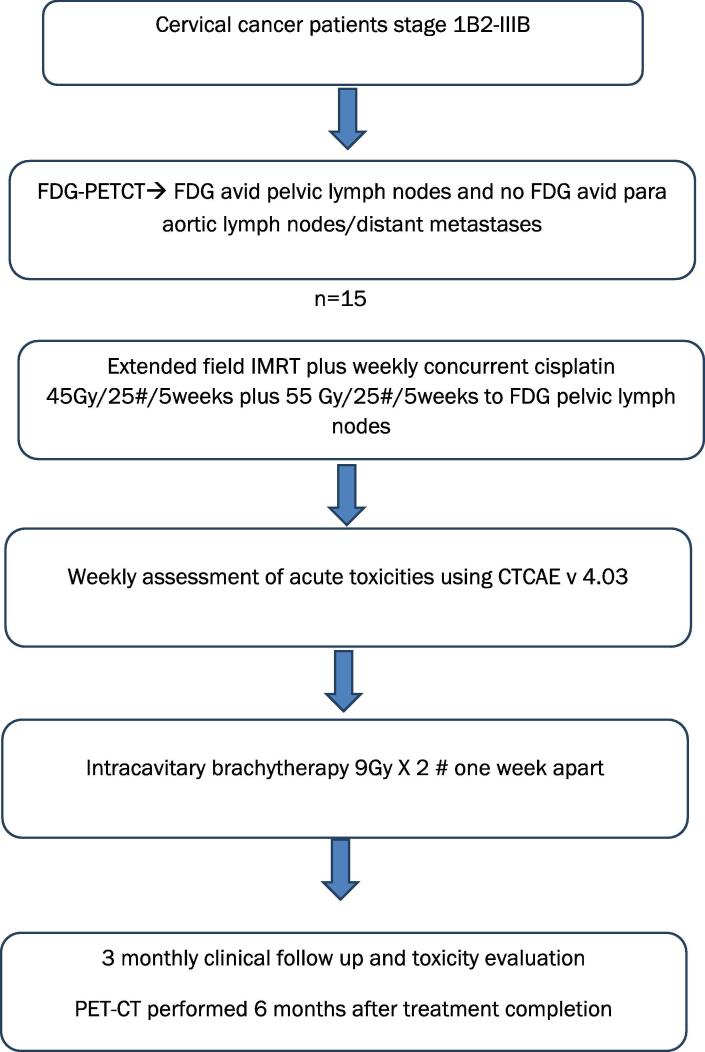


## Appendix A

STROBE Statement—checklist of items that should be included in reports of observational studies*Give information separately for cases and controls in case-control studies and, if applicable, for exposed and unexposed groups in cohort and cross-sectional studies.

**Note:** An Explanation and Elaboration article discusses each checklist item and gives methodological background and published examples of transparent reporting. The STROBE checklist is best used in conjunction with this article (freely available on the Web sites of PLoS Medicine at http://www.plosmedicine.org/, Annals of Internal Medicine at http://www.annals.org/, and Epidemiology at http://www.epidem.com/). Information on the STROBE Initiative is available at www.strobe-statement.org.
